# Psychosocial and mental health challenges facing perinatally HIV-infected adolescents along the Kenyan coast: a qualitative inquiry using the socioecological model

**DOI:** 10.3389/fpubh.2024.1379262

**Published:** 2024-07-23

**Authors:** Stanley W. Wanjala, Moses K. Nyongesa, Stanley Luchters, Amina Abubakar

**Affiliations:** ^1^Department of Public Health and Primary Care, Ghent University, Ghent, Belgium; ^2^Department of Social Sciences, Pwani University, Kilifi, Kenya; ^3^Neuroassessment Group, KEMRI/Wellcome Trust Research Programme, Centre for Geographic Medicine Research (Coast), Kilifi, Kenya; ^4^Institute for Human Development, Aga Khan University, Nairobi, Kenya; ^5^Centre for Sexual Health and HIV AIDS Research (CeSHHAR), Harare, Zimbabwe; ^6^Liverpool School of Tropical Medicine, Liverpool, United Kingdom; ^7^Department of Public Health, Pwani University, Kilifi, Kenya; ^8^Department of Psychiatry, University of Oxford, Oxford, United Kingdom

**Keywords:** perinatal HIV infection, adolescents, socioecological model, HIV-related stigma, qualitative inquiry, focus group discussion (FGD), H-Assessment

## Abstract

**Background:**

The advent of antiretroviral therapy has led perinatally HIV-infected (PHI) adolescents to live long, fulfilling lives through lifelong treatment. However, there is limited knowledge about the lived experiences and psychosocial and mental health challenges faced by PHI adolescents in sub-Saharan Africa, where 80% of PHI adolescents reside. To address this gap, we adapted the socioecological model to investigate the challenges and lived experiences of PHI adolescents in rural coastal Kenya.

**Methods:**

Between October and November 2018, a sample of 40 participants (20 PHI adolescents and their 20 primary caregivers) participated in a qualitative study using an H-assessment data collection approach for adolescents and focus group discussions with caregivers. Data analysis was conducted using a framework approach on NVIVO 11 software.

**Results:**

PHI adolescents from this setting experience many challenges across various levels of the ecosystem. At the individual level, challenges include living in denial, HIV status disclosure, antiretroviral adherence, internalized stigma, and mental health issues. Within the family, challenges such as parental loss, insufficient care from parents, and unacceptance lead to threats of harm. In the broader community, key challenges such as gossip, unsupportive community members, long waiting times at the health facility, isolation, rejection, and an unresponsive school system fail to address the needs of PHI adolescents. Finally, HIV-related stigma and discrimination manifested across different levels of the socioecological framework. To cope with these challenges, PHI adolescents often rely on privacy and social support from their families.

**Conclusion:**

The findings underscore the need to develop and implement multi-level adolescent-friendly interventions to address PHI adolescent challenges and guide future investment in adolescent’s health. Furthermore, there is a need to address internalized and interpersonal stigmas through individual-level interventions that promote resilience and the active involvement of adolescents, their caregivers, peers, and teachers who are their social support system.

## 1 Introduction

Adolescence, aged 10 to 19 years old as defined by the World Health Organization, represents a developmental and transitional phase between childhood and adulthood characterized by rapid growth and significant biological, psychological, and socioemotional transformations that support significant health implications (1–3). Furthermore, adolescence can be divided into early (10–14 years) and late (15–19 years) adolescence, each characterized by unique physical, emotional, cognitive, and social changes (4, 5). Over 16% of the world’s population are adolescents, emphasizing their pivotal role in achieving the 2030 sustainable development goals (1). Moreover, adolescents account for approximately 5% of all people living with HIV (PLWHA) and 11% of new HIV infections (6).

In 2022, an estimated 1.65 million adolescents were living with HIV infection across the world, the majority (85%) of them resided in sub-Saharan Africa (sSA) (6). Moreover, more than 80% of adolescents living with HIV (ALHIVs) acquired the infection through mother-to-child transmission (7). However, despite extensive efforts to prevent the vertical transmission of HIV, millions of children and adolescents in sSA remain perinatally infected (8). The majority of children infected perinatally with HIV are now in adolescence and early adulthood phases with a stigmatizing, potentially fatal, and chronic illness (9). Owing to the increased survival of ALHIVs due to antiretroviral therapy (ART) and vertically transmitted HIV among adolescents, the rate of increased survival continues to rise (10, 11). Relatedly, ALHIVs are the fastest-growing subgroup of PLWHA (11).

Living with HIV poses both psychosocial and socioeconomic challenges related to HIV and its care (12, 13). Particularly for PHI adolescents, the prolonged survival with HIV/AIDS (9) aggravates these challenges, impeding their ability to seek treatment and recommend for themselves (14, 15). The struggles these adolescents face extend beyond accepting their seropositive status and dealing with family members with positive status, and they struggle with the memories of deceased parents and uncertainties about their future, health, education, career, and marriage (16). Additionally, adolescence, marked by risk-taking and transitioning to greater independence, magnifies these challenges for adolescents. Parental influence diminishes risky behavior, which is prevalent, and the imperative to establish an identity and fit in with peers takes precedence (4, 5). Coping with a seropositive status becomes difficult during adolescence, necessitating a different management approach compared with adults.

The present study aimed at getting a nuanced understanding of the lived experiences and challenges faced by PHI adolescents. Understanding HIV-infected adolescents’ unique challenges is crucial in designing and developing effective interventions tailored to improve their quality of life. However, there is a shortage of data available on this population, and previous studies have mostly focused on quantitative research. While there are some existing qualitative studies on this topic, the data is still limited, and our study adds to the growing body of knowledge on this important topic by using an adaptation of the socio-ecological framework (17–20), which recognizes that the experience of health and illness transcends individual characteristics and examines the interplay between individual, interpersonal, community, and policy-level factors that shape the experiences of HIV-infected adolescents in a rural Kenyan setting. However, our study does not focus on the challenges at the public policy level. The socioecological model has been extensively applied in various fields, especially in public health, as it conceptualizes health broadly and focuses on the understanding how layered social environments impact individual behaviors and health outcomes. Since its conception in the 1970s, the socioecological model has been adapted to develop multilevel approaches in areas such as safe practices in primary care (21), violence prevention (22), immunization uptake (23), barriers to HIV clinic attendance (24), and psychosocial and mental challenges faced by people living with HIV (17).

Adolescents between 12 and 17 years old are typically belong to upper primary school to secondary school experience the same developmental phases. Compared with a wider age range of 10–19 years, including pre-adolescents and emerging adults who may be going through different life and developmental stages such as attending tertiary institutions or having a full-time job, adolescents aged 12–17 years share more homogenous emotional, cognitive, and social issues. This study was designed to explore challenges faced by PHI adolescents aged 12 to 17 years living with HIV in rural Kenya (at the intrapersonal/individual, interpersonal/family, and community levels) and understand the lived experiences of PHI African adolescents in a rural Kenyan setting.

## 2 Methods

We describe study methods and findings following the guidelines outlined in the Consolidated Criteria for Reporting Qualitative Research (COREQ) checklist (25) (see Supplementary Table S3). This checklist comprises 32 items that must be addressed to ensure explicit and comprehensive reporting of qualitative studies (25).

### 2.1 Study design and setting

In the context of HIV, this is a nested sub-study that is a part of baseline data collected from an ongoing larger longitudinal observational cohort study, the Adolescent Health Outcomes Study (AHOS), examining the neurocognitive and mental health outcomes among adolescents aged 12–17 years (26). The baseline data were collected between November 2017 and October 2018. The current study qualitatively examines the lived experiences and psychosocial and mental health challenges faced by PHI adolescents who participated in the baseline phase of the main study. This qualitative study was conducted at the Comprehensive Care and Research Clinic (CCRC) located in the Kilifi County Hospital, a public healthcare institution. The study was carried out by the Center for Geographic Medicine Research, Coast (CGMR-C), which is a part of the Kenya Medical Research Institute—Wellcome Trust Research Programme (KEMRI/WTRP) located in Kilifi County. Kilifi County is mostly a rural setting with an estimated population of 1.5 million, predominantly rural dwellers (27) with almost half of this population comprising individuals who are younger than 15 years old (28). Additionally, Kilifi County is among the poorest counties in Kenya (29), and the main economic activities of the residents of Kilifi County include subsistence farming, small-scale trading, and fishing (30). The county has low literacy levels and high rates of school dropouts (31).

### 2.2 Study participants and recruitment

In this sub-study, we recruited 20 PHI adolescents and their primary caregivers participating in the present study. These adolescents needed to be aware of their HIV status, receiving treatment at the CCRC. The caregivers were supposed to be aware of their HIV status and the primary carers of the PHI adolescents, willing to accompany them for assessments. Recruitment was carried out by a trained research assistant collaborating with experienced healthcare workers involved in the participating HIV treatment facilities. The emergence of data saturation (32, 33) determined the sample size during the data collection exercise in our study. The larger AHOS study recruited participants through consecutive sequential sampling from all families attending HIV clinic days at eight HIV treatment and care clinics in Kilifi County until the targeted number was achieved. The selection of the clinics was purposively done based on their distribution and client capacity of the HIV-specialized clinics in the Kilifi Health and Demographic Surveillance System (KHDSS) (30). In total, AHOS comprised 558 (201 perinatally HIV infected, 158 perinatally HIV exposed but uninfected, and 199 HIV unexposed and uninfected) adolescents. Further details about the AHOS study and its procedures have been described elsewhere (26).

### 2.3 Data collection materials and procedures

H-Assessments (Supplementary Table S1) were used to collect data from all adolescents, whereas Focus group discussions (FGDs) (Supplementary Table S2) were conducted on the caregivers of these adolescents. The H-assessment is pivotal for qualitative data collection as it engages respondents actively, not passively, and can be used to help children assess the strengths and weaknesses of their environment and the support they receive through pictures (34). Though ideal for children, the H-assessment can be adapted for adults. Using the H-assessment to begin a FGD with the adolescents gave them a chance to record their thoughts and experiences before discussing them with the larger group. FGDs were used to elicit responses from parents/caregivers about the lived experiences of PHI adolescents. In total, three H-assessments and three FGDs were held between October and November 2018.

After obtaining informed consent from caregivers and assent from adolescents, the FGDs and the H-assessments were audio-recorded. A trained facilitator and note-taker led the discussions in Kiswahili, one of the two national languages of Kenya and the most widely spoken language along the coast of Kenya. H-assessments lasting for 45–55 min were conducted on PHI adolescents by SWW and MKN in a spacious private room located in the neuro assessment offices at KEMRI. Furthermore, FGDs lasting for approximately 2 h were held with the caregivers of the PHI adolescents in a quiet spacious private room located in the neuro assessment offices at KEMRI. All interviews were conducted in Kiswahili, the official national language, and were recorded digitally after obtaining consent from all participants. The interviews were conducted by a team of researchers with different expertise: (1) a medical sociologist specialized in HIV/AIDS stigma (SWW), (2) an expert in mental health with expertise in global health (MKN), and (3) three trained fieldwork assessors (R.M., G.S., and A.C.) (see Supplementary Table S4). Facilitators of the FGDs and H-assessment used a semi-structured interview guideline containing open-ended questions to guide the discussions. The authors formed the interview guidelines with questions informed by the grounded theory. Separate interview guidelines were used for H-assessments on PHI adolescents and FGDs on their caregiver, and these interview guidelines were developed to delve deeply into stigma and discrimination challenges such as challenges experienced by PHI adolescents, their experiences of HIV stigma and discrimination and spaces where stigma takes place, and the perpetrators of stigma carrying toward PHI adolescents in the community.

### 2.4 Data management and analysis

Data were managed using NVivo qualitative data indexing software (version 11 Pro, QSR international). The audio-recorded interviews were transcribed verbatim, with all identifying information removed, translated into English, proofread, and uploaded to NVivo. Additionally, as described by Ritchie and Spice, data were analyzed using the framework approach (35), constantly applying comparative techniques (36) and resolving coding differences through mutual agreement. Codes were inductively generated by the analysis of transcripts by two researchers (SWW and MKN) and deductively generated by drawing on data from the interview guidelines.

An initial coding scheme was generated, expanded, and refined through additional coding against transcripts to incorporate emerging themes. Subsequently, data were summarized, categorized, and exported into matrices to compare themes systematically. Investigator triangulation (37) was used to ensure the credibility of the results (37), whereby two researchers (SWW and MKN) coded and analyzed the data. Regular meetings were held during data analysis to discuss emerging codes, and any discrepancies were resolved through discussion before updating the codebook. We achieved data saturation (32, 33), as sufficient information on our research question was collected.

### 2.5 Theoretical framework

The data analysis and reporting were based on an adaptation of the socioecological model (18, 19). This theory-based framework recognizes that health and illness experiences are frequently influenced by factors within and beyond an individual (18, 19). The application of this framework was intended to provide a robust platform for the investigation of challenges that PHI adolescents experience not only at the individual level but also at the interpersonal and community levels. This study aims to comprehend the challenges that PHI adolescents experience at the intrapersonal (individual) and interpersonal levels (family and community levels), as the latter covering the spaces in which PHI adolescents spend the maximum time (schools, healthcare facilities, and the playground). By using this framework and identifying the challenges occurring at multiple levels at which they occur, this study has the potential to provide recommendations that integrate health services to serve PHI adolescents more effectively.

### 2.6 Ethics statement

This study adhered to the ethical principles and guidelines for studies involving human participants as outlined in the Declaration of Helsinki. The local institutional review board, Scientific and Ethics Review Unit (SERU) of the Kenya Medical Research Institute (KEMRI), granted the ethical approval to recruit and interview participants (Ref SERU; KEMRI/SERU/CGMR-C/084/3454). Additionally, permission to work in the HIV care and treatment clinic was sought from and granted by the Department of Health, County government of Kilifi (Ref HP/KCHS/VOL.VIX/80). A legal caretaker collected data of all eligible adolescents at the CGMRC-KEMRI. Eligible adolescents provided written assent, whereas their caregiver/legal guardian provided written informed consent for their participation. Adolescents and caretakers were reimbursed for their travel costs depending on their residence.

## 3 Results

### 3.1 Participants’ characteristics

In this study, the analysis incorporated 6 transcripts, including 3 H-assessments with 20 PHI adolescents and 3 FGDs with 20 primary caregivers. The sociodemographic characteristics of the participants are presented in Table 1.

**Table 1:** Summary of participants’ socio-demographic characteristics.

Sample characteristics	Frequency (%)
**Socio-demographic characteristics**	
*PHI Adolescents (n = 20)*	
Age (12–17 years)	
Mean (SD)	14.35 (1.63)
Sex	
Female	8 (40%)
Male	12 (60%)
Number of years in school	
Mean (SD)	5.40 (2.35)
Religion	
Christianity	16 (80%)
Islam	4 (20%)
*Caregiver Participants (n = 20)*	
Age (32–56 years)	
Mean (SD)	44.35 (7.84)
Sex	
Female	16 (80%)
Male	4 (20%)
Education	
Primary	17 (85%)
Secondary	3 (15%)
Religion	
Christianity	16 (80%)
Islam	4 (20%)

### 3.2 Challenges faced by adolescents living with HIV

**Figure 1:**
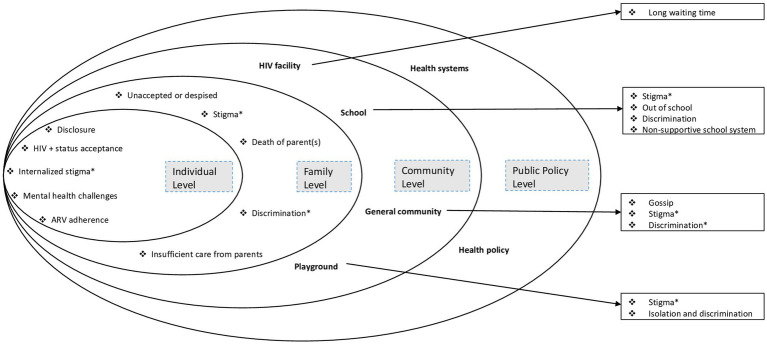
Challenges facing perinatally HIV-infected adolescents from coastal Kenya, mapped onto the socioecological model. In asterisk (*) are the inter-level challenges. Adapted from Nyongesa et al. (17).

Figure 1 presents an overview of the challenges faced by PHI adolescents in our setting, which is categorized according to various levels of the adapted socioecological system, including individual-level challenges, family-level challenges, and community-level challenges (general community, school, HIV clinic, and playground). Furthermore, this challenge illustrates both the emerging challenges specific to each level and overlaps across multiple levels within the socioecological system (see Figure 1).

#### 3.2.1 Individual-level challenges

##### 3.2.1.1 Acceptance of HIV-positive status

Caregivers and adolescents noted the challenges faced by adolescents living with HIV in accepting their positive HIV status. These challenges impacted their self-perception and overall well-being. Some adolescents may find it difficult to accept their HIV status, leading to a negative mindset and doubts about their ability to succeed in different areas of life. The constant reminder of their HIV status likely contributes to this negative self-perception, potentially affecting their motivation and confidence.

*If a PHI adolescent has not come to terms with their diagnosis, they might hold the belief that their efforts will not lead to success. Consequently, they consistently remind themselves of their HIV status, creating a perception that their goals are unattainable whenever they attempt to pursue a goal (Female HIV-infected adolescent, 13 years old).*

One participant shared their daughter’s experience, emphasizing the detrimental effects of living in denial on mental health, such as insanity indicating profound psychological distress. However, they emphasized the significance of accepting the HIV-positive status of an individual, receiving appropriate medical treatment, and adhering to antiretroviral (ARV) medications for maintaining the health and well-being of individuals living with HIV.

*Until last year but one, my daughter had been living in denial about her HIV status. Subsequently, her viral load was extremely high, and she eventually went ‘insane’ at some point. However, after she started adhering to her ARVs, she recently gave birth to a healthy baby (Female caregiver of an HIV-infected adolescent, 38 years old).*

##### 3.2.1.2 Disclosure of HIV status to others

Participants discussed how the disclosure of HIV-positive status had been a challenge because of the fear of being judged, too many questions (e.g., what the ARV medication was used to treat and why they carried it daily), the worry about the reactions they might face from people close to them, and fear of their HIV status spreading beyond those with whom they shared it.

*Moderator: You do not tell people at home that you are taking medicine. And why do not you tell them?*

*It’s your secret.*

*Moderator: What will they do if you tell them?*

*They will tell others (Female HIV-infected adolescent, 14 years old).*

*What will I tell my girlfriend if she discovers I am on ARVs? (Male HIV-infected adolescent, 15 years old).*

##### 3.2.1.3 Poor adherence to antiretroviral medication

PHI adolescents struggled to adhere to their prescribed medication regimen. They were involved in improper medication practices, such as taking ARVs at the wrong time, missing doses, and taking more than the prescribed or incorrect dosages. Managing ARV schedules, dealing with disclosure stigma, and the impact of non-disclosure on treatment adherence were additional challenges encountered by these adolescents.

*Taking ARVs in front of your peers is challenging because you are unsure how to do it discreetly without them noticing (Female HIV-infected adolescent, 14 years old).*

To avoid taking ARVs at a prescribed time during the day, some adolescents opted to take all the day’s ARVs once:

*Another challenge is overdosing. For instance, if the prescription is for two pills in the morning and two pills in the evening, he might inadvertently take all four at once, exceeding the recommended dosage. This could occur either in the morning or evening to eliminate the need to carry the ARVs to school (Male HIV-infected adolescents, 15 years old).*

The lack of disclosure of adolescents’ HIV-positive status to others created a dilemma for them as they were hesitant to take their ARVs in front of their classmates for fear of inadvertent disclosure. Consequently, this might result in skipping doses or stop taking ARVs.

*Adhering to ARV medication schedules is a challenge. If you carry your ARVs to school, you will face inquiries about the purpose of the ARVs and why you always have them every day. When faced with such situations, you will begin skipping doses or completely discontinue using your ARVs (Male HIV-infected adolescent, 13 years old).*

A caregiver suggested his child to carefully take the ARV at a water tap to avoid inadvertent disclosure of the HIV-positive status.

*The challenge he experiences is at school because his class schedule conflicts with his medication schedule. They leave for school at 6 am and return home at 8 pm. He is supposed to take his ARVs at 6 am and 6 pm. Sometimes he takes it (ARVs) around 8:30 pm when he arrives home from school. He is in a dilemma as he wants to take his ARVs but does not know how to do so without his friends knowing. I always advise him to take his ARVs at the tap so his peers think he is just getting water (Female caregiver of an HIV-infected adolescent, 49 years old).*

Despite adherence to medication practices proving to be a challenge, one adolescent expressed no fear in taking their ARVs and other prescribed drugs in front of other classmates. However, as a strategy to avoid stigma, discrimination, judgment, and unwanted attention associated with being HIV-positive, as well as to maintain a sense of privacy and protection from prejudice, they utilized the purpose of their medication by attributing it to a more socially acceptable disease, such as malaria.

*I do not fear taking my drugs in front of other people, when they ask what they are for I lie that they treat other diseases like malaria (Male HIV-infected adolescent, 16 years old).*

A participant described how disagreements or conflicts within a household, where the parents appeared indifferent or unconcerned about the situation, would potentially lead to an approach of an adolescent refusing to take their prescribed ARV medications, with severe consequences that could even lead to death.

*If disagreements occur at home and the parents seem like they do not care, it might lead to him refusing to take his meds which might lead to death (Female Parent of HIV-infected adolescent, 35 years old).*

##### 3.2.1.4 Mental health challenges

The death of parent(s) due to HIV/AIDS, stigma, and discrimination associated with HIV/AIDS, adolescents not accepting their positive HIV status, facility-level challenges such as long waiting times and inadvertent disclosure of HIV status may lead to adolescents experiencing common mental health problems, such as depression and anxiety. These mental health problems were expressed using some phrases such as “thinking too much” and “feeling sad,” which are similar to how these common mental health problems are understood locally. In addition, due to the fear of being judged or rejected by their peers, adolescents may isolate themselves and avoid social interactions. This self-imposed isolation can further exacerbate feelings of sadness, rumination (repetitive negative thinking), and self-loathing.

*This child was being discriminated against at home. His status was being disclosed to everyone in the community. As a result, the child was not at peace, he was thinking too much and isolated himself as he was uncomfortable associating with other people. He was filled with sadness, and constantly ruminated because he thought if he associated with his peers, he will labelled as someone suffering from HIV (Female caregiver of an HIV-infected adolescent, 34 years old).*

The psychological distress experienced by adolescents with HIV has detrimental effects on their lives. Some adolescents may stop following their antiretroviral medication while others may have suicidal thoughts. Excessive rumination results in difficulties concentrating in class, ultimately leading to poor academic performance.

*Due to constant rumination, a HIV-infected adolescent is likely to perform poorly in their exams. Additionally, discrimination at home and lack of enough personal time might lead to suicidal ideation as a means of finding solace from their worldly struggles (Male HIV-infected adolescent, 12 years old).*

*Discrimination might lead you to have suicidal thoughts (Female HIV-infected adolescent, 14 years).*

Another HIV-infected adolescent reported experiencing sadness when they take their ARVs after the prescribed time had lapsed because the person who usually reminds them to do so was not present.

*Maybe your parent has gone somewhere, and they are the ones who remind you when it is time to take your ARVs. If they return late past my time to take ARVs, I always feel sad because I am taking my drugs past the appointed time (Female HIV-Infected adolescent, 14 years old).*

##### 3.2.1.5 Internalized stigma

Participant narratives revealed that HIV-infected adolescents isolated themselves from peers due to a fear of transmitting the virus to them. In addition, the internalization of stigma revealed that adolescents may have concerns about potential discrimination, stigma, or negative treatment if their HIV status was known. Accordingly, adolescents preferred to remain their HIV-positive status a secret.

*We do not disclose our HIV positive status to others in the community because it is our secret (Female HIV-infected adolescent, 15 years old).*

*Interviewer: Does the teacher know that your child is living with HIV?*

*Participant: The teacher is unaware…my child told me, “I do not want my teacher to find out about my HIV status.” (Female caregiver of an HIV-infected adolescent, 36 years old).*

*Adolescents infected with HIV isolate themselves because they think if they interact with their peers, they might infect them (Female HIV-infected adolescent, 16 years old).*

#### 3.2.2 Family-level challenges

##### 3.2.2.1 Parental loss

A few participants mentioned that they were either full or partial orphans. Additionally, some participants revealed that the loss of parent(s) can be challenging to HIV-infected adolescents. Specifically, one HIV-infected adolescent can overcome by emotional distress following the loss of a parent.

*For example, if your mother or father has passed away, you are overcome with grief (Female HIV-infected adolescent, 14 years old).*

##### 3.2.2.2 Unacceptance leading to death threats

During the interviews, a participant described a scenario in which a group of adolescents contemplated killing of an HIV-infected adolescent (their cousin). This act was not only illegal but also morally abhorrent, driven by the fear of contracting the virus due to a lack of understanding about its transmission.

*In the homestead where I live, a lady died from AIDS and left a child with HIV. Some kids (cousins to the infected adolescent) in that homestead hatched a plan to kill him by mixing his food with rat poison. They feared they might contract the virus from interacting with him by playing or eating together. Fortunately, one of the children informed his father (uncle to the HIV-infected adolescent) about the plan, who then punished them for hatching such a plan. The uncle then sent the HIV-infected adolescent to live with an aunt in another town as his cousins had negative thoughts and feelings about him (Female caregiver of an HIV-infected adolescent, 38 years old).*

##### 3.2.2.3 Insufficient care from parents

According to a participant, adolescents living with HIV faced challenges such as unmet basic needs because their parent(s) were unable to provide adequate care. This necessitated committed caregivers to involve and fulfill the crucial caregiving role. In addition, siblings played an important role in recommending the well-being of adolescents living with HIV, as highlighted in the following quote:

*………. the kid used to live with his father since I am just a caregiver and not the parent. When my sister died, her husband had to care for the children. However, after some time, her siblings came to me and reported that their sister was suffering because when their father leaves, he does not leave food for her (Female caregiver of an HIV-infected adolescent, 45 years old).*

#### 3.2.3 Community-level challenges

##### 3.2.3.1 Health facility challenges

###### 3.2.3.1.1 Long waiting time

A caregiver of an HIV-infected adolescent expressed that long waiting times at the health facility posed a challenge. The adolescent consistently urged his mother to request for a prompt attention from the doctors, as he desired to go back to school and continue his education. If he did not attend quickly, he would become annoyed and angry due to missing out on lessons. The interruption in his education and the potential consequences on his academic advancement resulted in a significant frustration.

*……… My son always tells me to tell the doctors to attend to him quickly and give him his medication refills so that he can return to school and continue learning. As a result, if I accompany him to the clinic and he is not attended to promptly, he becomes irritable and angry because he has missed some lessons. However, if he is quickly attended to and returns to school, he is a happy boy because he will be able to study alongside other students (Female caregiver of an HIV-infected adolescent, 36 years old).*

##### 3.2.3.2 School environment challenges

Based on our interviews, it was found that school-going children faced institutional barriers when it came to following through with their medical appointments, particularly when they had to be absent from school to attend their clinic appointments.

###### 3.2.3.2.1 School system is unresponsive to the needs of HIV-infected adolescents

HIV-infected adolescents faced the difficulties of navigating an unresponsive school system that did not cater to their medical and educational needs. Before the teachers being informed of their HIV status, they consistently received punishment for their school absences when they had to go to the hospital for medical appointments. These adolescents encountered the challenge of regularly missing school whenever they needed to attend the clinic for their medical appointments. Other students reported difficulties in navigating issues how to coordinate with their medical appointments, especially when disclosure had not been made to the school authorities.

*Before the teacher knew what he was suffering from, he was always punished when he used to miss school because he had gone to the hospital for his ARV refill. However, I intervened and advised him to go and disclose his status to his class teacher and tell him that whenever he misses school, he is always at the hospital for his ARV refill. Nowadays, when he is not in school, they know he has gone to pick up his medication, and nobody punishes him as they know his status (Female caregiver of an HIV-infected adolescent, 38 years old).*

Other challenges at school included, among other things, non-consensual/inadvertent disclosure of status, missing classes for clinic appointments, experiencing discrimination and isolation at school (as discussed in the stigma section), and constantly being interrogated by their peers about the purpose of their medication and why they had to carry it with them to school daily.

*Other students always want to know why they are taking medication, and once they know, they refuse to sit close to them because of their HIV status (Female caregiver of an HIV-infected adolescent, 42 years old).*

The fear of unintended/non-consensual disclosure at school was a major concern for HIV-infected adolescents, especially the deterioration of their relationship with fellow students. HIV-related stigma and discrimination could extend beyond personal opinions and disagreements because even within a close friendship, knowledge of the adolescent’s HIV status might have been used as a means to exclude and socially isolate them.

*If you disagree with a friend who is aware of your HIV status at school, they might disclose your status to others and tell them not to interact with you because of your HIV status (Male HIV-infected adolescent, 15 years old).*

##### 3.2.3.3 Playground challenges

###### 3.2.3.3.1 Isolation and rejection

Some participants described experiences of frequently feeling unloved and being excluded from social activities, such as playing football during playtime, by their peers due to their HIV status. They were consistently instructed to play alone first and then promised for the inclusion, which led to the feelings of isolation and rejection.

*When people are playing football, they will refuse to play with me because of my HIV status, and thus they discriminate against me (Male HIV-infected adolescent, 16 years old).*

*They do not love me. When we are playing together, they always tell me to play alone then later; we will play together (Male HIV-infected adolescent, 14 years old).*

#### 3.2.4 Inter-level challenges

Various forms of HIV-related stigma (enacted, internalized, perceived, and anticipated) and discrimination against adolescents living with HIV were mentioned by the study participants at various levels of the socioecological system. HIV-related stigma was experienced at individual, family, and community levels (general community, school, and playground). HIV-related discrimination was mentioned at the family and community levels (general community, playground, and school). Table 2 presents the various forms of HIV-related stigma and discrimination experienced by these adolescents living with HIV.

**Table 2:** Types of HIV-related stigma and discrimination experienced by adolescents living with HIV from Kilifi, Kenya.

Level of social ecosystem	Forms of HIV–related stigma	Data sources	Select illustrative quote(s)	Forms of HIV discrimination	Data sources	Select illustrative quote(s)
Individual level	Internalized stigma	4 HIV-infected adolescents, 2 caregivers	*We do not disclose our HIV-positive status to others in the community because it is our secret (HIV-infected adolescent)* *I: Does the teacher know that your child is living with HIV?* *P: The teacher is unaware…my child told me, “I do not want my teacher to find out about my HIV status.” (Caregiver of an HIV-infected adolescent)* *What will I tell my girlfriend if she discovers I am on ARVs?(HIV-infected adolescent)* *When children get to know about their HIV status, they might self-isolate as they will think their peers will reject them. They isolate themselves and live like a lone ranger. (HIV-infected adolescent)*	–	–	–
Family level	Enacted stigma Perceived stigma	3 Perinatally HIV-infected adolescents	*When one wants to use the water closet seat, they are told not to use it as they can infect others who use the same toilet. A separate toilet will be constructed for their use (an HIV-infected adolescent)*	Isolation Parental neglect Denied food Separate utensils	6 Caregivers, 4 Perinatally HIV-infected adolescents	*They isolated him and did not want to eat together with him. They looked at him as a dog and gave him his own bowl. Initially, they used to eat together, but now they have been told that if he is given food, let him eat his food, and do not use or even touch them. (Caregiver of an HIV-infected adolescent)* *However, after some time, her siblings came to me and reported that their sister was suffering because when their father leaves, he does not leave food for her (Caregiver of an HIV-infected adolescent)*
Community level						
a) General community	Enacted stigma Perceived stigma	4 Perinatally HIV-infected adolescents, 4 Caregivers	*I went to the neighbour’s house and found them eating. The mother told me to excuse them for some time. Why is it that everywhere I go, I feel like I am being discriminated against (HIV-infected adolescent)* *Some neighbours warn their children against playing with HIV-infected adolescents because they might contract HIV through contact. (Caregiver of an HIV-infected adolescent)* *For example, when you pass near where your neighbours are seated, and they start gossiping about your HIV status (HIV-infected adolescent)*	Neighbour not wanting my child to sit next to her kids Denied food Friends refuse to associate with you once HIV status is known/Rejection	7 Caregivers, 5 Perinatally HIV-infected adolescents	*If my neighbour is serving her children food, then she sees my child, she will ask her what she wants and then send her back home, promising to share leftovers, but this is a ploy so that my child does not interact with her children (Caregiver of an HIV-infected adolescent)* *When our friends get to know of our HIV status, they might stop interacting with us as they will think that they can contract the virus through interaction or through sharing what we have with them (HIV-infected adolescent)* *Someone living with HIV is discriminated against by others in the community as they refuse to play with him and they cannot involve him in anything happening in the community (HIV-infected adolescent)*
b) Health facility			–	–	–	–
c) Within the school environment	Enacted stigma Perceived stigma Anticipated stigma	10 Perinatally HIV-infected adolescents 5 Primary caregivers	*If you disagree with a friend who is aware of your HIV status at school, they might disclose your status to others and tell them not to interact with you because of your HIV status (HIV-infected adolescent)* *When people start discussing HIV in school, you isolate yourself because you think they might mention you or a family member (HIV-infected adolescent)* *Whenever my child seeks permission from the teacher to go to the clinic, his peers always want to know where he is headed. Recently, a fellow student snatched his bag leading to his medicines spilling on the ground (caregiver of an HIV-infected adolescent)*	Other students are reluctant to sit next to an adolescent living with HIV/Isolation	2 Primary caregivers	*Other students always want to know why they are taking medication, and once they know, they refuse to sit close to them because of their HIV status (Caregiver of an HIV-infected adolescent)*
d) At the Playground	Enacted stigma Perceived stigma	4 Perinatally HIV-infected adolescents	*They do not love me. When we are playing together, they always tell me to play alone, then later; we will play together (HIV-infected adolescent)* *When people are playing football, they will refuse to play with me because of my HIV status, and thus they discriminate against me (HIV-infected adolescent)*	Isolation; peers avoiding contact with HIV-infected adolescents	3 Perinatally HIV-infected adolescents	*Our peers isolate us (PHI adolescents) and do not want to play with us who are infected (HIV-infected adolescents)*

At the family level, HIV-related discrimination was marked by insufficient care from parents, including denial of food, cessation of education, and isolation and separation of utensils.

*They isolated him and did not want to eat together with him. Relatives looked at him as a dog and gave him his own bowl. Initially, they used to eat together, but now they have been told that if he is given food, let him eat his food, and do not use or even touch them (Female caregiver of an HIV-infected adolescent, 36 years old).*

In the community, HIV-related discrimination resulted in the exclusion and isolation of HIV-infected adolescents, particularly when their peers became aware of their HIV status.

*When our peers become aware of our HIV status, they might stop interacting with us as they will think that they can contract the virus through interaction (HIV-infected adolescent).*

From our discussions, it became evident that both teachers and fellow students carried out HIV-related discrimination within the school environment. However, a parent warned the teacher about the potential consequences if her child were to complain again, implying that the teacher would face undesirable repercussions as a result.

*The challenge was that his peers and teachers gossiped about him having the virus (HIV), but I solved that. I warned the teacher that if it ever happened again, he would not like the consequences that would befall him. Right now, my child studies with no problem (Female caregiver of an HIV-infected adolescent, 40 years old).*

During our discussions with adolescents and their caregivers, we encountered various types of HIV-related stigma. At the family and community levels, participants discussed the presence of enacted and perceived stigma, while at an individual level, there was evidence of internalized HIV-related stigma (see Table 2 for selected excerpts). In the community, particularly on the playground, the manifestation of HIV stigma was observed as HIV-positive adolescents being excluded from playing with their peers solely because of their HIV status.

*When people are playing football, they will refuse to play with me because of my HIV status, and thus they discriminate against me (Male HIV-infected adolescent, 14 years old).*

### 3.3 Coping strategies

Although participants were not asked about the coping strategies they used for positive living, these adolescents mentioned several sources of support that aided their coping.

#### 3.3.1 Social support

Our findings suggest that HIV-related social support in this setting involved a wide range of supportive measures. In addition, practical assistance was mobilized through social support to promote medication adherence, which included reminders for adolescents to take their medication as prescribed and in handling school-related consequences, such as obtaining permissions for those adolescents who were unable to disclose their condition to the school authority (teachers).

*I had to talk to the teachers and the headmaster, explaining that my child needs to take medications. So if they notice he was absent from school, they should know that he had gone for his clinic appointment (Female caregiver of an HIV-infected adolescent, 53 years old).*

*Your parents are the ones who remind you when it is time to take your ARVs (Female HIV-Infected adolescent, 14 years old).*

#### 3.3.2 Secrecy

Respondents indicated that adolescents used various coping mechanisms, including HIV status concealment through either partial disclosure or complete non-disclosure. In one instance, a caregiver suggested to an adolescent that, to prevent their peers from discovering their status, they should discreetly take their medication from the water tap in school. Other participants chose to isolate themselves to avoid persistent inquiries about the purpose of their medication.

*He is in a dilemma as he wants to take his medication but does not know how to do so without his friends knowing. I always advise him to take his medication, go to the tap, and take it from there, as his peers would think he has just gone to drink water (Female caregiver of an HIV-infected adolescent, 36 years old).*

To maintain their medication routine while keeping their HIV status a secret, one participant chose to take their ARVs but resorted to deception by providing false information about the purpose of their medication. They attributed their medication intake to a more socially acceptable illness such as malaria, to conceal their actual HIV status. By doing so, they aimed to create the perception that their medication was for a less stigmatized condition.

*I do not fear taking my drugs in front of other people, when they ask what they are for I lie that they treat other diseases like malaria (Male HIV-infected adolescent, 15 years old).*

## 4 Discussion

### 4.1 Summary of key findings

This qualitative study aimed to gain a comprehensive and nuanced understanding of the day-to-day challenges encountered by PHI adolescents from rural coastal Kenya. We utilized an adaptation of the socioecological model as a framework to guide the analysis and reporting of these challenges. Various challenges emerged from the interviews with adolescents and their caregivers at different levels of the socioecological model. At the individual level, psychosocial and mental health issues emerged as significant challenges that greatly hampered the well-being and interaction of PHI adolescents. At the family level, participants mentioned challenges, such as death of parent(s), insufficient care from parents, and unacceptance leading to death threats. Within the general community, they experienced challenges, such as gossip and a lack of support from community members. Long waiting times at the HIV clinic was also mentioned as a challenge. Inflexible or strict school schedules and policies and disclosure to teachers led to absence in class for medical appointments, facing constant questioning from peers regarding the purpose of their appointments. Isolation and rejection by peers were mentioned as frequent challenges experienced by the adolescents within the playground. Finally, HIV-related stigma and discrimination in various forms were prevalent experiences for PHI adolescents across multiple levels of the socioecological framework. These negative attitudes and behaviors further aggravated the challenges they faced in their daily lives and affected their sense of belonging.

### 4.2 Comparison of study findings with previous research

Despite numerous studies documenting the psychosocial challenges affecting ALHIV (13, 38–41), there is a growing need to comprehend their lived experiences and contextual circumstances to address the obstacles hindering their uptake of HIV-related services and overall well-being (42), especially in resource-constrained settings. Our study builds upon the existing literature about challenges experienced by ALHIV by presenting a rich contextualized account of the psychosocial and mental health challenges faced by PHI adolescents in a resource-limited setting. The most common psychosocial challenges encountered by younger ALHIV (aged 12–19 years), as identified in a systematic review of qualitative studies from East Africa (13), include HIV-related stigma and discrimination in different manifestations, concerns regarding HIV disclosure, difficulties in adhering to ARV, and struggling with the implications of having an HIV-positive identity. In our study, in a similar study conducted within our setting among adolescents of the same age group (38), these challenges also emerged as frequent challenges faced by PHI adolescents.

The coping strategies utilized by respondents to positively cope with these challenges in the present study reflect what has been reported in previous studies (17, 39, 40, 43). For instance, social support can help adolescents adhere to their medication because peers who are aware of the status might remind them to take their medication. Similar to our findings, as reported in a previous study, caregivers were found to be a strong pillar of support and played an important role in navigating school permissions for students who were unable to face their teachers (44). Furthermore, disclosure by these adolescents can also help them get guidance on a healthy living (17). On the other hand, secrecy and describing as healthy as a coping strategy which impacted adherence to ART was used to avoid the inadvertent disclosure of their HIV status and subsequent stigmatization, especially in the school setting (38, 39).

In the absence of support through resilience strategies, stigma can manifest in different contexts, such as home or in school, having negative implications for the health outcome of adolescents (45). We found that experiences of internalized, enacted, perceived, and anticipated stigma were common in the daily life experiences of ALHIV in Kenya, similar to other studies (17, 38, 39). Anticipated stigma and the fear of rejection due to the internalization of stigma and the inevitability of enacted stigma which are pervasive in society were shown to influence ART adherence and disclosure to both peers and teachers within the school setting, a phenomenon that has been documented in other studies (17, 39). Enacted stigma was highlighted in respondents’ narratives of non-consensual disclosure by peers due to a disagreement, separation of utensils, bedding, sanitation facilities (toilet), and limited socialization within the home and school environments. This led to psychological distress, non-adherence to ART, and non-disclosure of HIV status.

The internalization of stigma and the negative inferences that respondents attributed to themselves coupled with misconception about HIV transmission led to the concealment of HIV status and non-adherence to ART similar to other studies (46). Self-imposed isolation due to internalized stigma have negative consequences on their mental health and social well-being through reduced social support, loneliness, and limited opportunities for social development. Finally, although not adversely mentioned, perceived stigma was evident in respondent narratives of peers who were advised against interacting with HIV-infected adolescents when HIV-infected adolescents were excluded from social activities. This limited their social interactions, resulting in a reduction in their support network. The outcomes of HIV-related stigma observed in this study are congruent with the findings from the previous research, which include psychological distress (38, 39, 47), reluctance to disclose HIV status (38, 48), suboptimal adherence to antiretroviral therapy (ART) (17, 49), and limited access to social support (39). HIV-infected adolescents lacking sufficient social support might face difficulties in building resilience, leading to challenges in adhering to ART and experiencing social isolation (45).

The anticipation of stigma coupled with the fear of constant questioning from peers about their medication, concerns about the spread of their HIV status beyond those they had shared with, and worries about the potential reactions from loved ones contributed to non-disclosure. Furthermore, our data suggest that the fear of disclosure contributes to sub-optimal adherence to ART, an issue that has been reported elsewhere (50). In addition, the timing of disclosure to adolescents by their caregivers has been shown to have detrimental effects on the adherence to medication as they questioned about the reason of taking the medication. Status disclosure, in the context of a chronic condition, has the potential to generate social support which has been demonstrated to play a crucial role in fostering resilience and motivating adherence (45) while also empowering adolescents to combat HIV-related stigma and actively engage in treatment support (51). Furthermore, it strengthens individual-level resilience factors including self-efficacy (51, 52), increasing self-confidence and motivation (52). Furthermore, research conducted in sSA demonstrates that disclosure to close family members and friends positively impacts the well-being and outcomes of ALHIV (53) but, if not rightly done, can lead to undesired outcomes.

Our findings revealed that medication adherence was a problem perceived by both caregivers and their PHI adolescents with some caregivers expressing challenges in ensuring medication adherence. Existing research suggests that interventions should prioritize addressing adolescents’ need for peer acceptance and social connection by teaching them effective disclosure methods to trusted individuals and providing guidance on navigating social situations (54). We propose interventions aimed at addressing interpersonal stigma that involves active engagement of both adolescents and individuals within their support system to address misconceptions and stigma while also enhancing psychosocial support and promoting adherence to treatment similar to recommendations in a previous study (55).

Even though not so many participants considered coming to terms with their HIV-positive status a challenge, others found this challenging as was mentioned by their caregivers. In the present study, the lack of acceptance about their HIV-positive status led them to have a perception which negatively impacts their motivation and self-confidence, resulting in a negative mindset and doubt about their ability to become successful life. Additionally, living in denial caused these adolescents psychological distress and contributed to sub-optimal adherence to ART. This could be attributed to the fact that they might have lost hope in life due to the chronic nature of HIV. Adolescents with an HIV-positive status including identity issues have been reported as a challenge in previous studies (17, 40, 43).

As reported before, within the sSA context, mental health issues are often expressed using local terms or idioms (56), necessitating the localized understanding (57) of mental health and illness for meaningful investigation (17). Our data suggest that the fear of judgment or rejection makes adolescents isolate themselves, avoiding social interactions and intensifying feelings of sadness, rumination, and self-hatred. Some participants used local idiomatic expressions to describe their experience of mental health issues such as anxiety and depression. In our study, mental health problems such as anxiety and depression that have been associated with suicidal tendencies and thoughts are similar to previous studies conducted among younger adolescents (aged 12–17 years) (38) and emerging adults (18–24 years) (17) in this setting. Furthermore, our findings reflect the conclusions drawn from a quantitative study indicating that mental health problems are prevalent among adolescents (58). It is worth noting that psychosocial issues contribute to mental health problems in PLWHA (17, 45).

At the family level, though not common, a participant reported that an adolescent who had not been accepted by peers due to the fear of HIV infection had considered being killed through poisoning. Unacceptance among emerging adults has been reported in a previous study (17). Other challenges that emerged within the family have previously been reported in other studies, such as parental neglect/insufficient care from parents and parental loss (17, 40, 43). While grieving following the loss of a loved one is a universal experience across different cultures (43), complications arise when prolonged grieving starts to have a detrimental effect on the individual’s life. The death of parents and extended periods of grieving among adolescents may be attributed to their anticipation of a bleak future in the absence of their primary caregivers. As with other studies, we recommend continued counseling support to assist these adolescents in effectively navigating the stages of grief (17).

Our findings that gossip and non-supportive community members are some of the community-level challenges reflect results from another study conducted in South Africa (43). In this study, HIV status disclosure as a result of gossip and rumor-mongering had the potential to contribute to stigma and discrimination. The non-supportive nature of community members deprives adolescents from the social support they require to navigate life with a chronic ailment. In line with previous studies conducted in Kenya (38, 44, 45, 49), Uganda (39), and South Africa (59), our analysis revealed that the school environment was notably unresponsive to the needs of PHI adolescents. Furthermore, our data suggest that stigma emerged as a significant obstacle faced by these adolescents in the school eco-system as they lacked a safe space to build community, disclose their status, and take their medication corroborating findings of a study conducted in Uganda (39). Participant narratives illuminate how some adolescents felt about teachers who were not particularly responsive to their needs as they were punished for school absences due to medical appointments.

Furthermore, the inflexibility of school schedules and policies and disclosure to teachers and PHI adolescents to adjust to when, where, and how often they took their medication to avoid inadvertently disclosing their HIV status were evident in our findings in case of the other studies carried out in sSA (39–44, 49, 60, 61). The experience of stigma has far-reaching consequences on the well-being of adolescents in the school, including their ART adherence and school engagement (44, 49). From our findings, as a result of stigma, some PHI adolescents admitted to being unable to concentrate in class and poor class participation. Poor concentration in class could lead to poor academic performance. Our findings suggest that psychosocial development of PHI, adolescents, e.g., their academic achievement may be compromised due to stigma. There is a need for future research to reduce HIV-related stigma within the school environment and mobilize social support apart from home; the school is also one of the most important socioecological contexts for well-being and development where adolescents spend most of their time.

Finally, our data indicated that the sentiments expressed by PHI adolescents regarding the playing field indicated the primary challenges they faced, such as feelings of isolation and rejection. Their attempts to join their peers in playing met the instructions to play alone with the promise of inclusion at a later time. Peers of PHI adolescents, possibly influenced by misconceptions or fear associated with HIV, expressed hesitation in engaging in close contact or playing with them. As a result, adolescents living with HIV experienced a sense of isolation and rejection as has been reported elsewhere (13, 38).

Stigma and discrimination continue to be significant factors in the daily life experiences of ALHIV. Despite four decades having passed since its emergence, the persistence of HIV stigma (62) still presents a perplexing challenge both for ALHIV and policymakers (38). Previous research has found that HIV-related stigma influences disclosure practices, uptake of HIV-related services, ART adherence, and social relationships and interactions (17, 38). Therefore, there is a need for the development of intervention initiatives specifically addressing HIV-related stigma, particularly among adolescents. A previous study among emerging adults living with HIV (17) emphasized the importance of considering the complex interplay between psychosocial issues when prioritizing HIV-related interventions. Our findings reported that struggling with an HIV-positive identity contributed to sub-optimal adherence to ART, negative self-perception, disclosure difficulties, and psychological distress among adolescents living with HIV. Therefore, it is crucial to recognize the interconnected nature of these factors when designing interventions to address psychosocial challenges faced by PHI adolescents.

### 4.3 Strengths and limitations of the study

This qualitative study used data triangulation to present a comprehensive analysis of the daily life experiences of PHI adolescents facing psychosocial and mental health challenges, offering in-depth insights into their thoughts and beliefs, as well as daily life experiences of their caregivers. However, several limitations should be taken into account when interpreting our findings. First, the study utilized a convenience sample consisting of PHI adolescents and their caregivers, which may restrict the generalizability of the results, although this is not atypical in qualitative research. Moreover, the perspectives gathered were specific to a population in rural coastal Kenya, and caution should be exercised when applying these findings to other regions in sSA or resource-limited settings. Finally, although we used the socioecological model, our study did not focus on the challenges at the public policy level.

## 5 Conclusion

Our findings indicated the fact that PHI adolescents experience several challenges at the individual, family, and community levels of the socioecological spectrum. Of note, HIV stigma and discrimination were manifested in various forms with far-reaching consequences at multiple levels of the socioecological framework. To effectively tackle these challenges and enable PHI adolescents to flourish and realize their full potential, it is crucial that stigma is understood and addressed at the different levels of the socioecological framework as it links directly to treatment adherence, disclosure, HIV status acceptance, and schooling. In addition, it is essential to develop and implement customized multi-level adolescent-friendly interventions accommodating the unique needs of adolescents. Additionally, these programmatic or policy interventions should incorporate relevant context-specific coping mechanisms and support structures to facilitate a smooth transition into adulthood. Finally, addressing internalized stigma necessitates individual-level interventions that promote resilience, while tackling interpersonal stigma requires the active involvement of adolescents and individuals in their support systems.

## Data availability statement

The raw data supporting the conclusions of this article will be made available by the authors, without undue reservation.

## Ethics statement

This study adhered to the ethical principles and guidelines for studies involving human participants as outlined in the Helsinki Declaration. The local institutional review board, Scientific and Ethics Review Unit (SERU) of the Kenya Medical Research Institute (KEMRI) granted the ethical approval to recruit and interview participants (Ref SERU; KEMRI/SERU/CGMR-C/084/3454). Additionally, permission to work in the HIV care and treatment clinic was sought from and granted by the Department of Health, County government of Kilifi (Ref HP/KCHS/VOL.VIX/80). All eligible adolescents were accompanied by a legal caretaker for data collection at the CGMRC-KEMRI. Eligible adolescents provided written assent, whereas their caregiver/legal guardian provided written informed consent for their participation. Adolescents and caretakers were reimbursed for their travel costs depending on their residence.

## Author contributions

SW: Conceptualization, Data curation, Formal analysis, Investigation, Methodology, Project administration, Software, Supervision, Visualization, Writing – original draft, Writing – review & editing. MN: Conceptualization, Data curation, Formal analysis, Methodology, Project administration, Software, Supervision, Writing – review & editing. SL: Conceptualization, Formal analysis, Methodology, Supervision, Validation, Writing – review & editing. AA: Conceptualization, Data curation, Formal analysis, Funding acquisition, Methodology, Resources, Software, Supervision, Validation, Writing – review & editing.
