# Supplementary material for: Psychosocial and mental health challenges facing perinatally HIV-infected adolescents along the Kenyan coast: a qualitative inquiry using the socioecological model
Source: Front Public Health. 2024 Jul 23;12:1379262. doi: 10.3389/fpubh.2024.1379262 (PMC11300237; doi:10.3389/fpubh.2024.1379262)
Supplement: Supplementary file 1 [file Table_1.docx]

Supplementary Material

**Psychosocial and Mental Health Challenges Facing Perinatally HIV infected Adolescents Along the Kenyan Coast: A Qualitative Inquiry Using the Socio-ecological Model**

**Stanley W. Wanjala^1, 2*^, Moses K. Nyongesa^3, 4^, Stanley Luchters^1, 5, 6^, Amina Abubakar^3, 4, 7, 8^**

*** Correspondence:** Stanley W. Wanjala; Amina Abubakar
s.wanjala@pu.ac.ke; amina.abubakar@aku.edu

Supplemental Table 1**:** H-Assessment

**H Assessment with Adolescent Representatives of Community – Methodology**

| **Activity** | **Objective** | **Details** | **Document** |
| --- | --- | --- | --- |
| **H Assessment** | Discuss factors that fight against or propagate stigma against adolescents and identify space-based indicators of environments that propagate or fight against stigma meted out on adolescents. | Identify key spaces in which adolescents spend time.  For each space, discuss with adolescents:   - How do you feel when you are in this space? Does this space propagate or fight against stigma meted out on adolescents? - What are the characteristics, people, or things in this space that propagate or fight against stigma meted out on adolescents living with HIV? (Probe for factors relating to the physical, social, and emotional environment in each space.) - How does this space (including people, activities, and things in it) affect girls? - How could this space be improved?   Use the H assessment for each space as appropriate   \| Factors that fight stigma against adolescents  ☺ \| Name of space \| Factors that propagate stigma meted out on adolescents  ☹ \| \| --- \| --- \| --- \| \| Suggestions for improvement \|   Ask adolescents more general questions:   - Are there traditions or practices in your community fight against or propagate stigma meted out on adolescent’s? How do these traditions or practices fight against or propagate stigma? - Who in the community influences or has power over factors that fight against or propagate stigma on adolescents?   Use the H assessment for each space as appropriate   \| Practices that fight against stigma on adolescents  ☺ \| Overall community \| Practices that propagate stigma on adolescents  ☹ \| \| --- \| --- \| --- \| \| Suggestions for improvement \| | - Detailed notes on the activity and discussion – including factors propagating and factors fighting stigma against adolescents, and suggestions for improvement. - For each space, labeled photos with descriptions. - Photographs of H assessments on flipcharts. |

**H Assessment with Adolescent Representatives of Community – Notes on Discussion**

|  |  | | | |
| --- | --- | --- | --- | --- |
| Community/School: |  | | | |
| Village: |  | | | |
| Facilitators: |  | | | |
| Date: |  | | | |
| Start time: |  | | | |
| End time: |  | | | |
| Group: | Adolescents  (13-17 years old) | Girls  Boys  Mixed | Includes adolescents with disabilities?  Includes adolescents from marginalized groups? | Number of participants:  ___ female  ___ male |
| Facilitator Notes: |  | | | |

| Can you tell me some of the challenges that you encounter as adolescents living with HIV/AIDS?  Can you tell me what you understand by the term stigma?  What are some of the indicators of stigma?  Who stigmatizes adolescents living with HIV/AIDS?  Can you tell me whether there is a time you willingly refused to participate in an activity because of your HIV status?  What are the effects of stigma on adolescent’s adherence to medication?  What are the effects of stigma on adolescent’s mental health?  What are the effects of stigma on adolescent’s physical health?  Can you tell me what disclosure is? | |
| --- | --- |
| What makes adolescents happy?  What makes adolescents achieve their goals? | |
| What do adolescents do to cope when people stigmatize them? | |
| How did you get to know/ find out about your HIV status?  Who told you about your HIV status?  What were your reactions to this kind of news?  Have you ever disclosed your HIV status to anyone? If yes, Who?  How do you think not disclosing your HIV status affects your physical health?  How do you think not disclosing your HIV status affects your mental health?  How do you think not disclosing your HIV status affects your adherence to medication? | |
| In which spaces do adolescents spend most of their times? | |
| **Name of Space (1) Home** |  |
| **Overall Comments** |  |
| **Factors that support adolescents living with HIV** |  |
| **Factors that harm adolescents living with HIV** |  |
| **Effects of stigma on Adolescents adherence to medication** |  |
| **Effects of stigma on Adolescents mental health** |  |
| **Effects of stigma on Adolescents Physical health** |  |
| **Impacts of stigma on disclosure practices** |  |
| **Benefits of disclosure** |  |
| **Effects of Disclosure on adolescents** |  |
| **Effects of non-disclosure on adolescents physical health** |  |
| **Effects of non-disclosure on adolescents mental health** |  |
| **Effects of non-disclosure on adolescents medication adherence** |  |

| **Name of Space (2) School** |  |
| --- | --- |
| **Overall Comments** |  |
| **Factors that support adolescents living with HIV** |  |
| **Factors that harm adolescents living with HIV** |  |
| **Effects of stigma on Adolescents adherence to medication** |  |
| **Effects of stigma on Adolescents mental health** |  |
| **Effects of stigma on Adolescents Physical health** |  |
| **Impacts of stigma on disclosure practices** |  |
| **Benefits of disclosure** |  |
| **Effects of Disclosure on adolescents** |  |
| **Effects of non-disclosure on adolescents physical health** |  |
| **Effects of non-disclosure on adolescents mental health** |  |
| **Effects of non-disclosure on adolescents medication adherence** |  |

| **Name of Space (3) Healthcare centers** |  |
| --- | --- |
| **Overall Comments** |  |
| **Factors that support adolescents living with HIV** |  |
| **Factors that harm adolescents living with HIV** |  |
| **Effects of stigma on Adolescents adherence to medication** |  |
| **Effects of stigma on Adolescents mental health** |  |
| **Effects of stigma on Adolescents Physical health** |  |
| **Impacts of stigma on disclosure practices** |  |
| **Benefits of disclosure** |  |
| **Effects of Disclosure on adolescents** |  |
| **Effects of non-disclosure on adolescents physical health** |  |
| **Effects of non-disclosure on adolescents mental health** |  |
| **Effects of non-disclosure on adolescents medication adherence** |  |

| **Overall Community** |  |
| --- | --- |
| **Factors that support adolescents living with HIV** |  |
| **Factors that harm adolescents living with HIV** |  |
| **Effects of stigma on Adolescents adherence to medication** |  |
| **Effects of stigma on Adolescents mental health** |  |
| **Effects of stigma on Adolescents Physical health** |  |
| **Impacts of stigma on disclosure practices** |  |
| **Benefits of disclosure** |  |
| **Effects of Disclosure on adolescents** |  |
| **Effects of non-disclosure on adolescents physical health** |  |
| **Effects of non-disclosure on adolescents mental health** |  |
| **Effects of non-disclosure on adolescents medication adherence** |  |

**
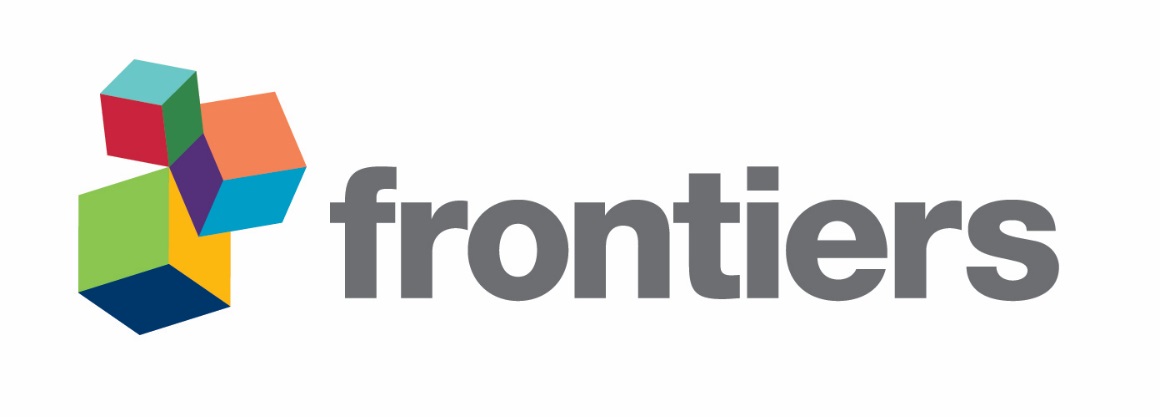
**
