# Supplementary material for: Psychosocial and mental health challenges facing perinatally HIV-infected adolescents along the Kenyan coast: a qualitative inquiry using the socioecological model
Source: Front Public Health. 2024 Jul 23;12:1379262. doi: 10.3389/fpubh.2024.1379262 (PMC11300237; doi:10.3389/fpubh.2024.1379262)
Supplement: Supplementary file 2 [file Table_2.docx]

Supplementary Material

**Psychosocial and Mental Health Challenges Facing Perinatally HIV Infected Adolescents Along the Kenyan Coast: A Qualitative Inquiry Using the Socio-ecological Model**

**Stanley W. Wanjala^1, 2*^, Moses K. Nyongesa^3, 4^, Stanley Luchters^1, 5, 6^, Amina Abubakar^3, 4, 7, 8^**

*** Correspondence:** Stanley W. Wanjala; Amina Abubakar
s.wanjala@pu.ac.ke; amina.abubakar@aku.edu

Supplemental Table 2. Focus Group Discussion (FGD) guide

| - | **Greet** the participants |
| --- | --- |
| - | **Introduce** yourself |
| - | Give **background information** about the study. Carefully read through the informed consent form and answer any questions. Give **information about the interview** |
| - | Assure that the participants that they **do not have to participate** if he/she does not want to |
| - | Ask for **approval** to participate in the interview |
| - | Tell the participants that **he/she can stop** the interview any time they wish |
| - | Assure **confidentiality** |
| - | Make sure the participants know what the tape-recording procedure is. Ask for **approval**. |
| - | Ask if the participant has any **questions** before the interview |
| - | If the participant agrees to take part please ensure they sign the consent form |
| - | If the participant agrees to take part please ensure they sign two copies of the consent form, give them a copy and keep one copy for our records. |
| - | Check **the tape recording equipment** |
| - | Start the interview |
| - | At the start of the interview, do NOT record *respondents name or any identifier*; just indicate ***the date, time, gender age and educational level of the person being interviewed. Lastly, record where the interview was taking place.*** |

1. What are the challenges experienced by adolescents (children aged 12-17 years) living with HIV in Kilifi?
2. I would like us now to discuss in more detail issues surrounding stigma.

- What do you understand by the term stigma?
- Drawing from your experiences and those of your children, what are some of the indicators of stigma?
- Can you share some examples of places where stigmatization of adolescents takes place?
- Can you share some examples (but do not mention names of persons involved) of people who stigmatize adolescents living with HIV? (Probe for what happened in this places to qualify them to classify the action as stigmatizing)
- As parents/caregivers of adolescents living with HIV, how do you think stigma affects adolescent’s adherence to their medication?
- As parents/caregivers of adolescents living with HIV, how do you think stigma affects adolescent’s mental health?
- As parents/caregivers of adolescents living with HIV, how do you think stigma affects adolescent’s physical health?

1. I would now like us to discuss issues related to disclosure.

- How did the disclosure process occur to your child? Who disclosed to your child about their HIV status?
- What was their reaction to this kind of news?
- Whom have you talked to about your child’s or your families’ HIV status? (Probe for why they decided to talk to another person about their children or family’s HIV status)
- Is there a reason why you do not tell some people about your child’s or your families HIV status?
- Do you think not disclosing about your child’s status has an impact on their functioning;
- Impact on health
- Impact on medical adherence
- Impact on social relationships
- Impact on education

**
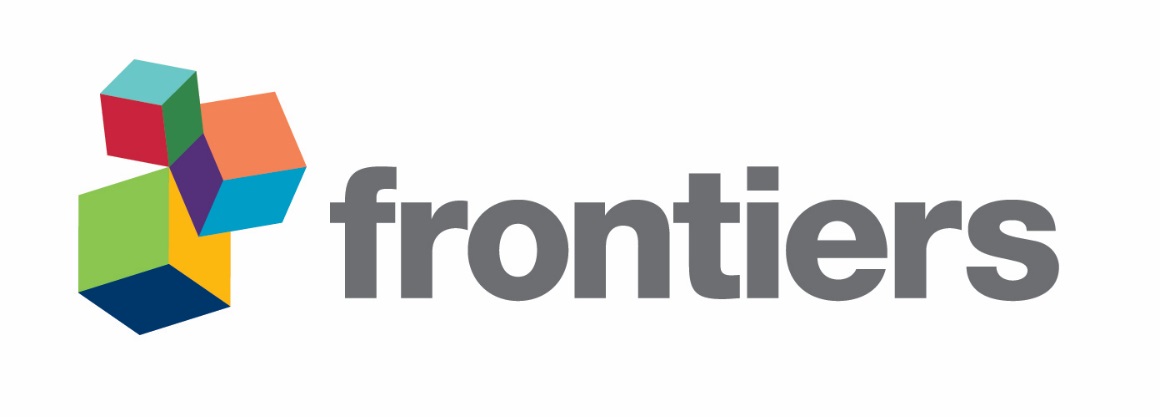
**
