# Supplementary material for: Psychosocial and mental health challenges facing perinatally HIV-infected adolescents along the Kenyan coast: a qualitative inquiry using the socioecological model
Source: Front Public Health. 2024 Jul 23;12:1379262. doi: 10.3389/fpubh.2024.1379262 (PMC11300237; doi:10.3389/fpubh.2024.1379262)
Supplement: Supplementary file 3 [file Table_3.docx]

Supplementary Material

**Psychosocial and Mental Health Challenges Facing Perinatally HIV Infected Adolescents Along the Kenyan Coast: A Qualitative Inquiry Using the Socio-ecological Model**

**Stanley W. Wanjala^1, 2*^, Moses K. Nyongesa^3, 4^, Stanley Luchters^1, 5, 6^, Amina Abubakar^3, 4, 7, 8^**

*** Correspondence:** Stanley W. Wanjala; Amina Abubakar
s.wanjala@pu.ac.ke; amina.abubakar@aku.edu

Supplemental Table 3. CORE-Q (COnsolidated criteria for REporting Qualitative research) Checklist

| Item No. | Topic | Guide Questions/Description | Reported on Page No. |
| --- | --- | --- | --- |
| **Domain 1: Research team and reflexivity** | | | |
| *Personal Characteristics* | | | |
| 1 | Interviewer/facilitator | Which author/s conducted the interview or focus group? *Data from H-assessments and FGDs are presented in this manuscript.* | Page 5,  Supplemental Table 1 and Supplemental Table 2 |
| 2 | Credentials | What were the researcher’s credentials? *The manuscript presents interviewer-facilitated data; thus, we describe the credentials of the researchers who participated in this study.* | Page 6, Supplemental Table 4 |
| 3 | Occupation | What was their occupation at the time of the study? *We describe the occupations of the researchers on the study team at the time of the study.* | Page 6,  Supplemental Table 4 |
| 4 | Gender | Was the researcher male or female? *The genders and initials of all the researchers who participated in each stage of the study team are described for our readers to make a connection between the individuals and names within the authorship.* | Pages 6,  Supplemental Table 4 |
| 5 | Experience and training | What experience or training did the researcher have? *We describe both the experience and training of each researcher on the study team.* | Supplemental Table 4 |
| *Relationship with participants* | | | |
| 6 | Relationship established | Was a relationship established prior to study commencement? *Researchers elucidated the study objectives to potential participants and informed them of their right to decline participation or withdraw consent at any research stage. Adolescents provided written assent whereas their caregivers/legal guardians provided written informed consent for their participation in the study* | Page 7 |
| 7 | Participant knowledge of the interviewer | What did the participants know about the researcher? (e.g., personal goals, reasons for doing the research) *Researchers engaged with participants with respect to audio-recording of HIV-related stigma conversations. Potential participants were informed about the study objectives and their right to decline participation or withdraw consent at any research stage.* | Page 7 |
| 8 | Interviewer characteristics | What characteristics were reported about the interviewer/facilitator? e.g., bias, assumptions, reasons and interest in the research topic? *Potential participants were acquainted with the study objectives and their right to decline participation in the study or withdraw consent at any research stage without any consequence.* | Page 7 |
| **Domain 2: Study design** | | | |
| *Theoretical framework* | | | |
| 9 | Methodological orientation and theory | What methodological orientation was stated to underpin the study? e.g., grounded theory, discourse analysis, ethnography, phenomenology, content analysis. *We employed the framework analysis in this study.* | Pages 6 |
| *Participant selection* | | | |
| 10 | Sampling | How were participants selected? e.g., purposive, convenience, consecutive, snowball. *We used a convenience sample selected study participants depending on availability and willingness to participate.* | Page 5 |
| 11 | Method of approach | How were participants approached? e.g., face-to-face, telephone, mail, email. *Participants were mainly recruited by a trained research assistant collaborating with experienced healthcare worker attached to the participating HIV treatment facilities.* | Page 5 |
| 12 | Sample size | How many participants were in the study? *There was a total of 40 qualitative respondents.* | Page 1, Table 1 |
| 13 | Non-participation | How many people refused to participate or dropped out? Reasons? *Participation in the H-assessments and FGDs was voluntary and data related to refusal to participate or drop out is unknown.* | n/a |
| *Setting* | | | |
| 14 | Setting of data collection | Where was the data collected? e.g., home, clinic, workplace. *Participants were interviewed in a quiet spacious private room located within the neuro assessment offices at the Kenya Medical Research Institute (KEMRI)* | Page 4 |
| 15 | Presence of non-participants | Was anyone else present besides the participants and researchers? *No one else was present besides the researcher and participants.* | Page 5-6 |
| 16 | Description of sample | What are the important characteristics of the sample? e.g., demographic data, date. *Participants’ socio-demographic data are presented in Table 1.* | Table 1 |
| *Data collection* | | | |
| 17 | Interview guide | Were questions, prompts, guides provided by the authors? Was it pilot tested? *The H-assessment and FGD guide and probes are provided.* | Supplemental Table 1 and Supplemental Table 2 |
| 18 | Repeat interviews | Were repeat interviews carried out? If yes, how many? *There were no repeat interviews carried out in this study.* | n/a |
| 19 | Audio/visual recording | Did the research use audio or visual recording to collect the data? *Recorders were used to audio-record interviews, which were later transcribed verbatim, translated into English, reviewed for accuracy, and uploaded on Nvivo.* | Page 6 |
| 20 | Field notes | Were field notes made during and/or after the interview or focus group? *n/a* | n/a |
| 21 | Duration | What was the duration of the interview or focus group? *The H-assessments lasted between 45-55 minutes with the FGDs lating lasting around two hours* | Page 5-6 |
| 22 | Data saturation | Was data saturation discussed? *The emergence of data saturation which was used to determine the sample size during the data collection exercise was discussed by the researchers* | Page 5 |
| 23 | Transcripts returned | Were transcripts returned to participants for comment and/or correction? *n/a* | n/a |
| *Data analysis* | | | |
| 24 | Number of data coders | How many data coders coded the data? *We provide information describing the number, role and identification of all data coders for this study.* | Page 5-6,  Supplemental Table 4 |
| 25 | Description of the coding tree | Did authors provide a description of the coding tree or codebook? *no* |  |
| 26 | Derivation of themes | Were themes identified in advance or derived from the data? *Themes were inductively derived from raw data and deductively by drawing on questions from the FGD.* | Page 6 |
| 27 | Software | What software, if applicable, was used to manage data? *We used NVIVO 11 software.* | Page 6 |
| 28 | Participant checking | Did participants provide feedback on the findings? *We did not consent patients/caregivers to provide feedback on findings.* | n/a |
| *Reporting* | | | |
| 29 | Quotations presented | Were participant quotations presented to illustrate the themes/findings? Was each quotation identified? e.g., participant number. *Representative quotes are embedded within the text* | Pages 8-18 |
| 30 | Data and findings consistent | Was there consistency between the data presented and the findings? *We demonstrate consistency between the data presented in the Results section and the interpretation of findings in the Discussion section.* | Pages 7-23 |
| 31 | Clarity of major themes | Were major themes clearly presented in the findings? *We presented all major themes in detail.* | Pages 7-23, Figure 1 |
| 32 | Clarity of minor themes | Is there a description of diverse cases or discussion of minor themes? *We provide a variety of rich quotes within the text.* | Pages 7-23 |

Adapted from: Tong A, Sainsbury P, Craig J. Consolidated criteria for reporting qualitative research (COREQ): a 32-item checklist for interviews and focus groups. *International Journal for Quality in Health Care*. 2007. Volume 19, Number 6: pp. 349 – 357.https://doi.org/10.1093/intqhc/mzm042

**
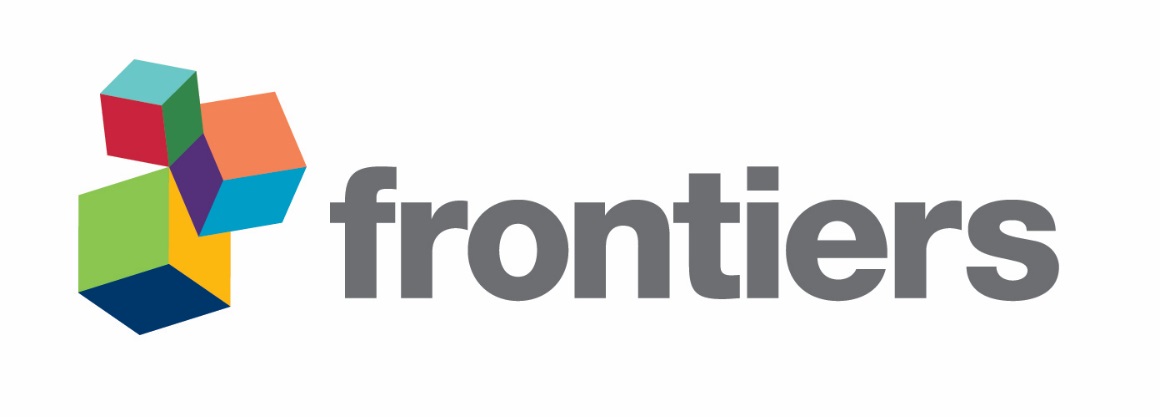
**
