# Supplementary material for: Psychosocial and mental health challenges facing perinatally HIV-infected adolescents along the Kenyan coast: a qualitative inquiry using the socioecological model
Source: Front Public Health. 2024 Jul 23;12:1379262. doi: 10.3389/fpubh.2024.1379262 (PMC11300237; doi:10.3389/fpubh.2024.1379262)
Supplement: Supplementary file 4 [file Table_4.docx]

Supplementary Material

**Psychosocial and Mental Health Challenges Facing Perinatally HIV infected Adolescents Along the Kenyan Coast: A Qualitative Inquiry Using the Socio-ecological Model**

**Stanley W. Wanjala^1, 2*^, Moses K. Nyongesa^3, 4^, Stanley Luchters^1, 5, 6^, Amina Abubakar^3, 4, 7, 8^**

*** Correspondence:** Stanley W. Wanjala; Amina Abubakar
s.wanjala@pu.ac.ke; amina.abubakar@aku.edu

Supplemental Table 4**:** Research Team Attributes and Qualifications

| **Author** | Attributes and Qualifications |
| --- | --- |
| S.W.W. | A Kenyan male social scientist with a Master of Arts in Medical Sociology, a Bachelor of Arts degree in Sociology, qualitative research methods training, and global health training. His research interests include HIV-related stigma as well as maternal and child health. |
| M.K.N. | A trained nurse with a Doctorate in Global Mental Health, a Master’s in Global Mental Health, a Diploma in Health Research Methods, and qualitative research training. He is interested in disease comorbidity research intersecting between psychiatric disorders and HIV/AIDS and the testing of psychological interventions seeking to address mental disorders comorbid with HIV using m-health platforms. |
| S.L. | He holds a medical degree, an MSc in Public Health for Developing Countries, and a Ph.D. in Health Sciences. His work has centred on sexual and reproductive health and maternal and child health. Within this field, he has advanced several focus areas, including the Involvement of men in improving maternal and neonatal health outcomes, the effects of climate change on maternal and child health, Sexually transmitted infections, and key populations such as sex workers and people who inject drugs. |
| A.A | She is a Developmental Psychologist with a Ph.D. in the neurodevelopmental assessment of children in rural sub-Saharan Africa. Abubakar’s thematic focus is on children at risk of experiencing developmental delays due to exposure to various health problems, particularly HIV, malnutrition, and malaria. Her research interests include quantifying the neurocognitive burden of early childhood diseases, developing culturally appropriate psychological measures for use in SSA, and identifying culturally appropriate intervention strategies for at-risk children in SSA. |
| R.M | Works as a field worker at the Kenya Medical Research Institute/Wellcome Trust Research Programme, Centre for Geographic Medicine Research (Coast), Kilifi, Kenya. She is a team member in the neuro assessment group. She is responsible for identifying and recruiting study participants and data collection. |

**
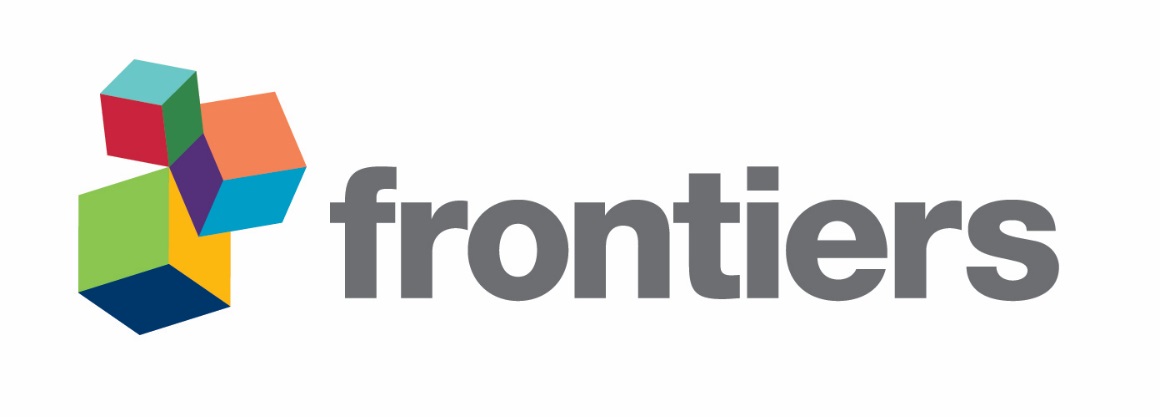
**
